# 17β-Estradiol Induced Sex Reversal and Gonadal Transcriptome Analysis in the Oriental River Prawn (*Macrobrachium nipponense*): Mechanisms, Pathways, and Potential Harm

**DOI:** 10.3390/ijms24108481

**Published:** 2023-05-09

**Authors:** Pengfei Cai, Huwei Yuan, Zijian Gao, Hui Qiao, Wenyi Zhang, Sufei Jiang, Yiwei Xiong, Yongsheng Gong, Yan Wu, Shubo Jin, Hongtuo Fu

**Affiliations:** 1 Wuxi Fisheries College, Nanjing Agricultural University, Wuxi 214081, China; ckgg5436@126.com (P.C.); yuan08102021@126.com (H.Y.); gaozijiangenomics@163.com (Z.G.); 2 Key Laboratory of Freshwater Fisheries and Germplasm Resources Utilization, Ministry of Agriculture and Rural Affairs, Freshwater Fisheries Research Center, Chinese Academy of Fishery Sciences, Wuxi 214081, China; qiaoh@ffrc.cn (H.Q.); zhangwy@ffrc.cn (W.Z.); jiangsf@ffrc.cn (S.J.); xiongyw@ffrc.cn (Y.X.); gongys@ffrc.cn (Y.G.); wuy@ffrc.cn (Y.W.)

**Keywords:** 17β-Estradiol, histological observations, *Macrobrachium nipponense*, sex ratio, sex reversal, transcriptome

## Abstract

Sex reversal induced by 17β-estradiol (E_2_) has shown the potential possibility for monoculture technology development. The present study aimed to determine whether dietary supplementation with different concentrations of E_2_ could induce sex reversal in *M. nipponense*, and select the sex-related genes by performing the gonadal transcriptome analysis of normal male (M), normal female (FM), sex-reversed male prawns (RM), and unreversed male prawns (NRM). Histology, transcriptome analysis, and qPCR were performed to compare differences in gonad development, key metabolic pathways, and genes. Compared with the control, after 40 days, feeding E_2_ with 200 mg/kg at PL25 (PL: post-larvae developmental stage) resulted in the highest sex ratio (female: male) of 2.22:1. Histological observations demonstrated the co-existence of testis and ovaries in the same prawn. Male prawns from the NRM group exhibited slower testis development without mature sperm. RNA sequencing revealed 3702 differentially expressed genes (DEGs) between M vs. FM, 3111 between M vs. RM, and 4978 between FM vs. NRM. Retinol metabolism and nucleotide excision repair pathways were identified as the key pathways for sex reversal and sperm maturation, respectively. Sperm gelatinase (*SG*) was not screened in M vs. NRM, corroborating the results of the slice D. In M vs. RM, reproduction-related genes such as cathepsin C (*CatC*), heat shock protein cognate (*HSP*), double-sex (*Dsx*), and gonadotropin-releasing hormone receptor (*GnRH*) were expressed differently from the other two groups, indicating that these are involved in the process of sex reversal. Exogenous E_2_ can induce sex reversal, providing valuable evidence for the establishment of monoculture in this species.

## 1. Introduction

Steroid hormones, mostly found in the testes, ovaries, hepatopancreas, and hemolymph, play significant roles in the regulation of gonadal development, sex determination, and growth in aquatic species through their interactions with endocrine factors [1]. Among these hormones, 17β-Estradiol (E_2_), primarily associated with female reproductive function including the growth and development of the ovaries and uterus [2,3], has been extensively studied in many species. Obtaining female individuals of sexual differentiation is easiest and most conveniently performed by including E_2_ in diets [4]. A study on Atlantic halibut (*Hippoglossus hippoglossus* L.) revealed that the addition of E_2_ treatment yielded a 70–74% female population. In a recent study on brown trout (*Salmo trutta*), the 20 and 30 mg/kg treatment groups of estradiol resulted in 84 and 86% female populations, respectively, much higher than the 47% for the control group after 456 days of feeding [5]. In Atlantic cod (*Gadus morhua*), the proportion of males was significantly reduced by adding 10 and 20 mg/kg of E_2_ to the diet [6].

The oriental river prawn (*Macrobrachium nipponense*) is a species of crustacean widely distributed in China, Japan, Korea, Vietnam, and Myanmar [7,8]. However, during the reproductive period, adult female *M. nipponense* ovaries mature rapidly and periodically, leading to a significant decline in the market specifications of females. This issue, combined with high stocking densities and environmental deterioration, can ultimately affect the entire harvest yield. One promising approach is using E_2_ to artificially induce sex reversal and develop reliable monoculture technology. Crustaceans can be more susceptible to sex reversal when exposed to testosterone during early development as they are lower aquatic animals. Disturbance of testosterone during this stage could result in sex reversal [9]. A report on narrow-clawed crayfish *(Astacus leptodactylus*) showed that E_2_ injection to females, bath to pleopodal eggs, and bath to stage 1, 2, and 3 juveniles increased the feminization rate significantly compared to the control [10]. A similar study in freshwater prawn (*Macrobrachium rosenbergii*) suggested that the use of E_2_ for sex reversal in prawns should be approached with caution. After the developmentally sensitive period that causes sexual differentiation has passed, it may be difficult for E_2_ to cause sex reversal [11].

Some studies have reported that most hormones are released into the water through waste or metabolism in less than 72 h [3,12]. Despite the potential benefits of E_2_, excessive quantities can cause harm to the animal, such as damage to organs [13], abnormal development [14], cancer risk [15], etc. A study investigating the effects of E_2_ on the liver and kidneys of male mice found that exposure to high levels of E_2_ caused damage to both organs [16]. A review article summarized the potential effects of E_2_ on behavior in animals and concluded that high levels of E_2_ can lead to changes in aggression, mate selection, and other behaviors. To our knowledge, there are no studies on long-term exposure or consumption of high levels of the hormone in this species.

In the present experiment, we successfully reversed the sex of crustaceans using E_2_ for the first time and established the gonadal transcriptome of neo-males (RM) and unreversed male prawns (NRM). The objectives of this study were to (1) determine whether dietary supplementation with different concentrations of E_2_ could induce sex reversal in *M. nipponense*; (2) compare histological and transcriptional differences in the gonads of neo-males to reveal the mechanics of E2-induced sex reversal in males, and key metabolic pathways and genes; (3) compare histological and transcriptional differences in the testes of unreversed male prawns to reveal the damage of E_2_ on male organs and the potential harm. The results of this study provide important evidence and techniques for achieving sex reversal in *M. nipponense* and reveal damage to males from excessive E_2_.

## 2. Results

### 2.1. Effects of Different Concentrations of E_2_ of Juvenile Prawns

#### 2.1.1. Sex Ratio

**Figure 1:**
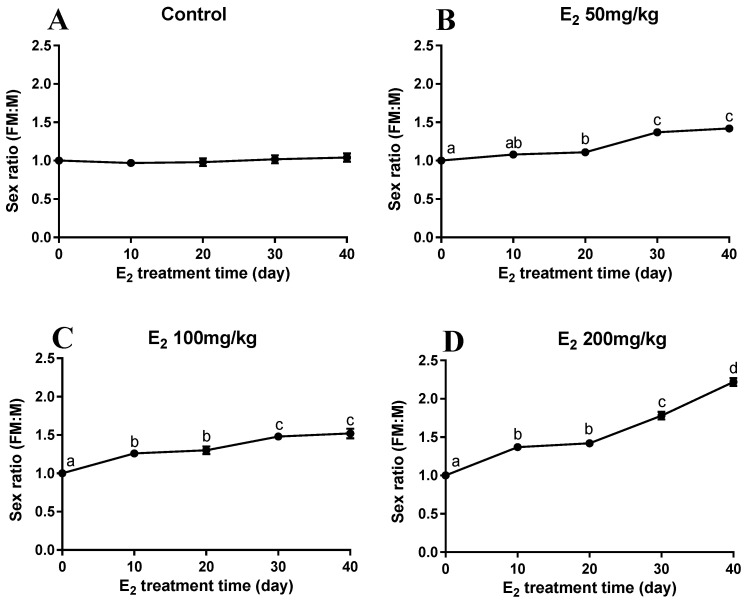
The sex ratio at four concentrations of 17β-estradiol(E_2_) over time. (**A**): Control; (**B**): 50 mg/kg E_2_; (**C**): 100 mg/kg E_2_; (**D**): 200 mg/kg E_2_. Data are shown as mean ± SEM of tissues from separate individuals (n = 3). Lowercase letters indicate differences in expression between different samples in the same group.

Figure 1 showed four concentrations of E_2_ over various lengths of time for the sex ratio (female:male). As shown in Figure 1A, throughout the culture phase the sex ratio of the control group was maintained around 1 (*p* > 0.05). Compared with the control group, the sex ratios all increased in the experiment groups. The best group was 200 mg/kg in Figure 1D, with a sex ratio of 2.22 at day 40, and was significantly different (*p* < 0.05).

#### 2.1.2. Histological Observations of the Gonad

**Figure 2:**
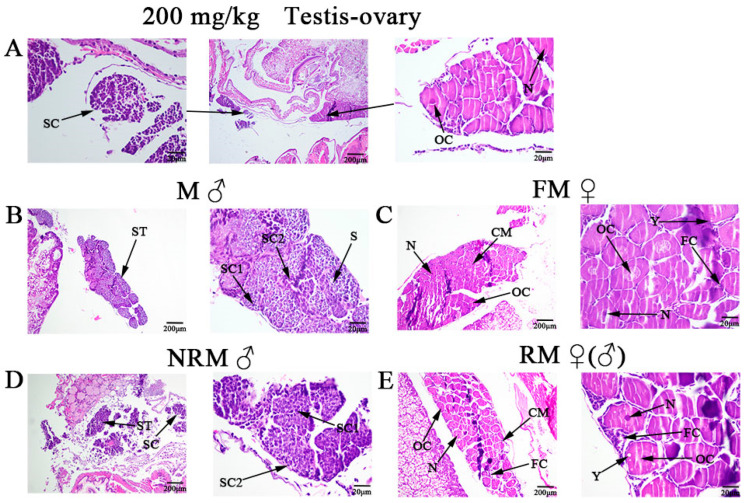
Histological sections of (**A**) testis-ovary of male prawns in the 200 mg/kg E_2_. (**B**) Testis histological section of male prawns. (**C**) Ovary histological section of female prawns. (**D**) Testis histological section of unsex-reversed male prawns. (**E**) Ovary histological section of neo-male prawns. N: Nucleus; ST: Spermatid; SC: Spermatocyte; SC1: Primary spermatocyte; SC2: Second spermatocyte; S: Sperm; OC: Ovarian cavity; FC: Follicle cells; CM: Cytoplasmic membrane; Y: Yolk granule. Scale bars: 200 µm and 20 µm.

Figure 2A showed gonadal sections of female prawn from the 200 mg/kg experimental group after 40 days, in which the co-existence of testis-ovary was observed, spermatocyte filled in testis and nucleus can be seen in the ovarian cavity. Figure 2B,C were the gonadal sections of male and female prawns. Figure 2D was a testis section of male prawn that was unsex-reversed fed at 200 mg/kg E_2_, and Figure 2E was an ovary section of female prawn that sex-reversed. Compared with Figure 2B, there were no mature spermatozoa in the slower-developing testis in Figure 2D. However, Figure 2C,E showed that neo-male prawn ovaries developed normally, with many oogonia, primary oocytes and cytoplasmic membranes observed and yolk granule accumulation.

### 2.2. The Comparative Transcriptomic Analysis

#### 2.2.1. Overview of Transcriptome Sequencing

After the low-quality reads were filtered out using FastQC, the following data were obtained for the four groups: 6,708,932,325 clean reads for M; 6,300,505,500 for FM; 7,237,298,550 for RM; 6,769,141,350 for NRM (Table 1). It was observed that the Q20 values of all samples, as detected by FastQC26, exceeded 95%, indicating high sequencing quality. All sequencing reads were stored in the Short Read Archive (SRA) of the National Center for Biotechnology Information (NCBI), and available with the accession number PRJNA961994. The first step in annotating all assembled unigenes involved querying the Nr (non-redundant) database. A total of 27,304 unigenes were successfully annotated in the Nr database. However, further investigation is required to determine the function of 4355 unannotated unigenes with novel genes.

#### 2.2.2. Identification and Functional Analysis of DEGs

**Figure 3:**
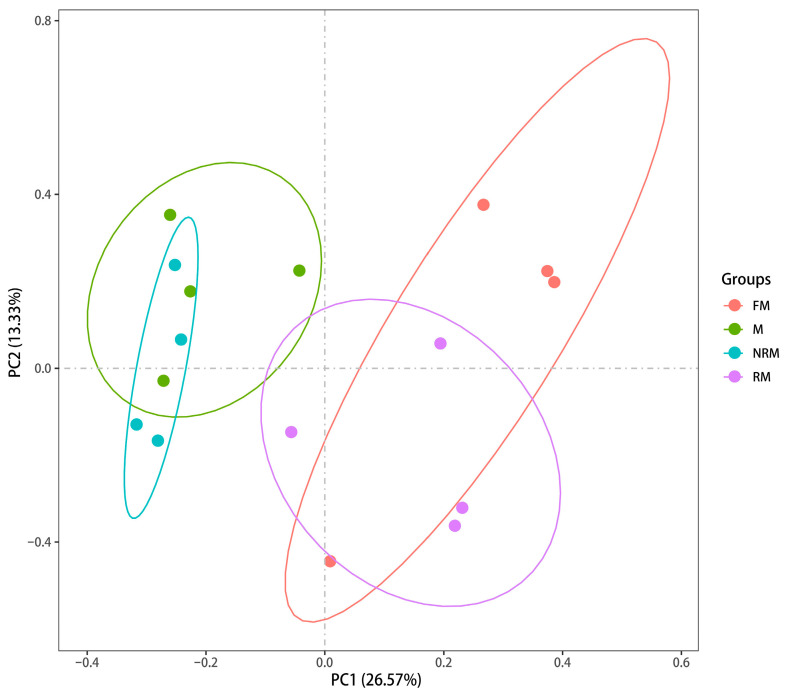
PC1 and PC2 represent the first and second principal components, and the percentages in parentheses represent the contribution of the first principal component to the sample variance.

Principal components analysis (PCA) was used to calculate the correlation coefficient between different samples and differentiate them from one another. The resulting PCA clustering can be seen in Figure 3. These analyses provide a powerful tool for understanding the relationships between different samples and their underlying gene expression profiles. During the analysis, the original *p*-values resulting from hypothesis testing were corrected using the widely accepted and effective Benjamini–Hochberg method. This approach allowed for the reduction in the False Discovery Rate (FDR) when screening differentially expressed genes by calculating adjusted *p*-values. The screening criteria used to identify significant DEGs were FC ≥ 2 and FDR ≤ 0.01.

**Figure 4:**
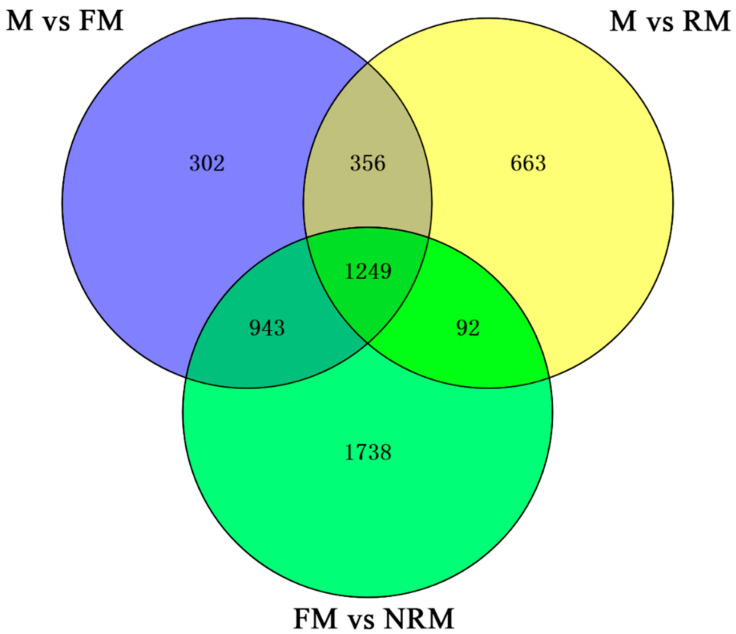
Analysis of DEGs by Venn diagram showing the number of DEGs in M vs. FM, M vs. RM, and FM vs. NRM comparisons.

The analysis results revealed a total of 3702 DEGs in the “M vs. FM” comparison, with 2569 upregulated genes and 1133 were downregulated. Similarly, the “M vs. RM” comparison identified 3111 DEGs, consisting of 2062 upregulated genes and 1049 downregulated genes. Additionally, the “FM vs. NRM” comparison showed 4978 DEGs, with 1579 upregulated genes and 3399 downregulated genes. These findings provide valuable insights into the gene expression changes occurring between different experimental groups (Figure 4).

#### 2.2.3. GO and COG Enrichment Analysis of DEGs

**Figure 5:**
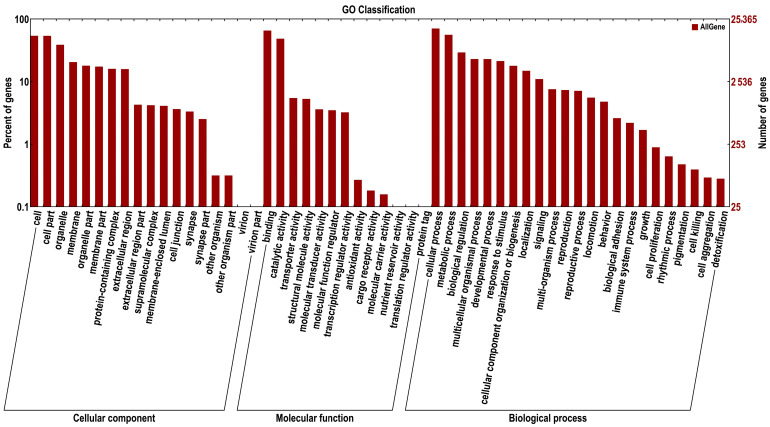
GO classification of unigenes; the abscissa is the second-level term under the three categories of GO. The ordinate represents the number of genes annotated to the term and the percentage of all genes.

**Figure 6:**
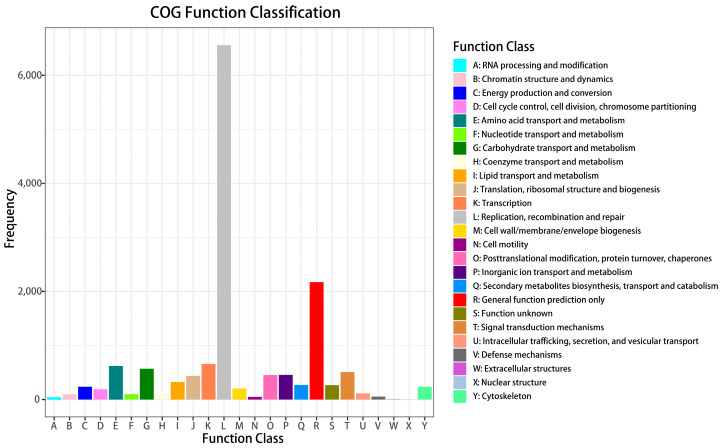
Clusters of orthologous groups of proteins (COG) classification of putative proteins.

Gene products were clustered by the GO and COG databases to describe their functional attributes. A total of 25,365 unigenes matched the known proteins in the GO database and were clustered into cellular components, molecular functions, and biological processes (Figure 5). The majority of cellular components were represented by cell (13,692 unigenes), cell part (13,666 unigenes), and organelle (9795 unigenes). The majority of molecular functions were represented by binding (16,621 unigenes) and catalytic activity (12,340 unigenes). The majority of biological processes were represented by cellular processes (17,897 unigenes) and metabolic processes (14,197 unigenes). A total of 12,255 unigenes were assigned to the matched proteins in the COG database and included 23 functional categories (Figure 6). Replication, recombination, and repair annotated the largest number of unigenes (6116 unigenes), followed by General function prediction only (1401 unigenes).

#### 2.2.4. KEGG Analysis and Important Differentially Expressed Pathways

**Figure 7:**
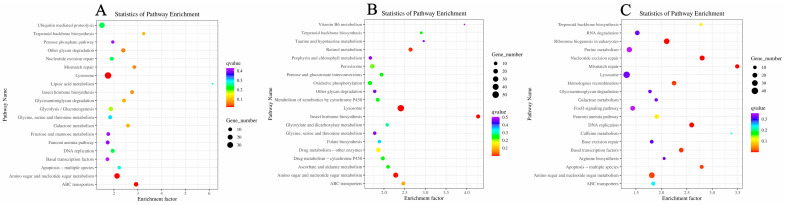
KEGG enrichment of DEGs. (**A**) M vs. FM, (**B**) M vs. RM, (**C**) FM vs. NRM.

KEGG analysis was applied to identify the biological pathways related to the unigenes. There were 509 DEGs mapped to 122 pathways in the comparisons of M vs. FM (Figure 7A). Among them, nine pathways including ABC transporters, Lysosome, Amino sugar and nucleotide sugar metabolism, other glycan degradation, Mismatch repair, Insect hormone biosynthesis, Terpenoid backbone biosynthesis, Galactose metabolism, and Glycosaminoglycan degradation in Table 2 (q-value < 0.1). Similarly, 475 DEGs were enriched in 121 pathways in the comparison of M vs. RM (Figure 7B). Five pathways, including Lysosome, Insect hormone biosynthesis, Amino sugar and nucleotide sugar metabolism, Retinol metabolism, and ABC transporters, with significant differences are shown in Table 2 (q-value < 0.1). There were 514 DEGs annotated in 121 pathways in the comparison of FM and NRM (Figure 7C). Seven pathways with significant differences including Nucleotide excision repair, DNA replication, Ribosome biogenesis in eukaryotes, Basal transcription factors, Apoptosis—multiple species, Homologous recombination, and Amino sugar and nucleotide sugar metabolism in Table 2 (q-value < 0.1).

#### 2.2.5. The Response of Reproduction-Related Genes to Sex Reversal

In the M/FM, M/RM, and FM/NRM comparisons, a total of 25 reproduction-related genes were screened. As shown in Table 3, the genes upregulated in male and female prawns are cyclin B, cystatin, cathepsin B, VASA-like protein, vitellogenin, vitellogenin receptor, ferritin, Fem1b, feminization-1, gametocyte-specific factor and gonadotropin-releasing hormone receptor, the downregulated genes are sperm gelatinase, doublesex and mab-3 related transcription factor, Kazal-type protease inhibitor, chitinase 3C, chitinase 1B, chitinase 3A and male reproductive-related protein. Some additional genes that occurred in M/RM and were downregulated included cathepsin C, cathepsin L, heat shock protein cognate, doublesex, and legumain-like protein. This suggests that they may be involved in the process of sex reversal. Compared with M/FM and M/RM, some genes were specifically expressed such as sperm gelatinase, cathepsin B, cathepsin L, legumain-like protein, peritrophin, and gustavus.

#### 2.2.6. Validation of DEGs by qRT-PCR

**Figure 8:**
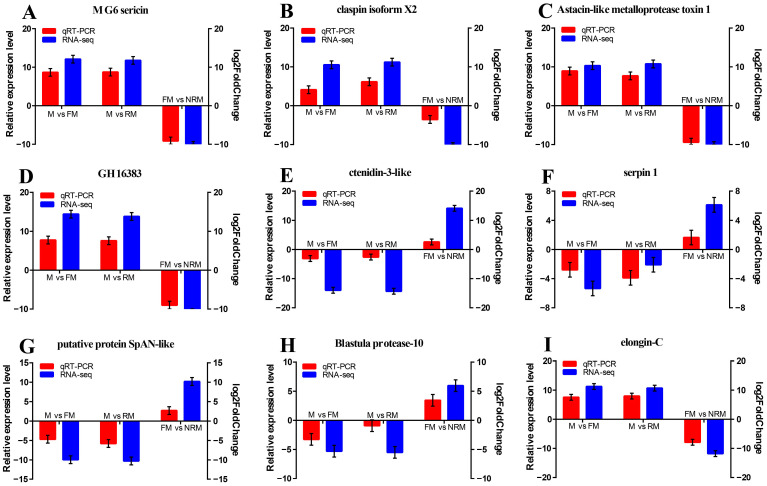
qRT-PCR validation of RNA-Seq data. (**A**) MG6 sericin; (**B**) claspin isoform X2; (**C**) Astacin-like metalloprotease toxin 1; (**D**) GH16383; (**E**) ctenidin-3-like; (**F**) serpin 1; (**G**) putative protein SpAN-like; (**H**) Blastula protease-10; (**I**) elongin-C. The left *Y*-axis represents the relative expression level determined by qRT-PCR, and the right *Y*-axis represents log2FoldChange determined by RNA-Seq. Data are shown as mean ± SEM of tissues from separate individuals (n = 3).

To validate the transcriptome results, nine DEGs were selected randomly that showed significantly different expression levels for qRT-PCR analysis in Figure 7. Positive numbers represent an upward trend and negative numbers represent a downward trend. As shown in Figure 8, the expression patterns of the nine DEGs identified by qRT-PCR were generally similar to those obtained in the RNA-Seq analyses. Although the relative expression levels were not completely consistent, this confirms that the current transcriptome sequencing data are reliable.

## 3. Discussion

This study aimed to determine whether dietary supplementation with different concentrations of E_2_ could induce sex reversal in oriental river prawns. The regulatory effect of E_2_ feeding on sex differentiation was analyzed by transcriptome analysis of sex-reversed prawn gonads. In this experiment, more females were observed after feeding E_2_ to post-larval juvenile *M. nipponense* in 50, 100, and 200 mg/kg. After 40 days, feeding E_2_ at a concentration of 200 mg/kg at the PL25 (PL: post-larvae developmental stage) resulted in the highest sex ratio (female: male) of 2.22:1. Histological observations also demonstrated the co-existence of testis and ovaries in the 200 mg/kg group. Furthermore, some male prawns at the PL30 did not reverse sex (NRM) after being fed 200 mg/kg E_2_, while others were reversed (RM) into females. Compared to male prawns, the testis developed slowly in NRM without mature sperm and spermatogonia were in the primary stage. Compared to female prawns, yolk granule accumulation, primary oocytes, and cytoplasmic membrane were observed in RM. The study indicated that vertebrate sexual hormones could induce sex reversal in crustaceans and determined that neo-males (sex-reversed male prawns) could be obtained by feeding 200 mg/kg E_2_ at the PL30 developmental stage. Interestingly, a high survival rate of *M. nipponense* was observed during and after hormone treatment, with even males without sex reversal remaining alive and eating normally. However, other studies have reported increased mortality for fish-fed hormone-treated feed [5,17,18]. The difference in these studies may be due to the concentration of E_2_ or the fact that *M. nipponense* are invertebrates and have a different excretion pattern from fish. While the feminization of male *M. nipponense* was successfully achieved in this study, the sex ratio did not reach 100%. One speculation is that the efficiency of sex reversal may be influenced by environmental factors such as water temperature and climate [19]. Further research may be required, such as extending the feed period or increasing the concentration of hormones.

In this study, a total of 69,545 transcripts were obtained, providing insight into the changes in transcriptional regulation within the gonads during sex reversal and helping to understand the molecular mechanisms of sex differentiation and gonad development in this species. According to the GO and COG analyses, genes related to male sexual development were predicted to be mainly found in the functional groups of cellular process, binding, metabolic process, and cells in the GO assignment, and in the functional groups of replication, recombination and repair, general function prediction only, and transcription in the COG classification. The number of DEGs between M vs. FM, M vs. RM, and FM vs. NRM was 3702, 3111, and 4978, respectively, indicating that supplementation of 200 mg/kg E_2_ in the diets affected the gonadal development of male prawns and more genes were activated in unsex-reversed males. This is consistent with histological observations of the RM and NRM. qPCR verification of nine randomly selected DEGs showed the same expression pattern as RNA-Seq, indicating the accuracy of the RNA-Seq.

Based on KEGG analysis comparing M vs. RM with M vs. FM, the Retinol metabolism pathway may play an important role in the sexual differentiation of *M. nipponense*. Retinoids (vitamin A) are critical to most forms of life. In chordates, they play an important role in the control of cell differentiation, regulation of immune competence, and reproduction during embryogenesis and in the adult organism. In both animals and humans, too high or too low levels of retinoic acid (RA) induce significant pathological changes during development. Retinoids primarily act by binding to retinoic acid receptors on DNA. During development, *Cyp26a1* and *Cyp26b1* play major roles in establishing RA gradients and regulating the differentiation of various stem cells [20]. In a recent study in mice, RA was found to promote the expression of some ovarian markers and inhibit the expression of some testis expressions. Furthermore, the absence of *cyp26b1* leads to impaired steroidogenesis and feminization of the reproductive tract [21]. This is consistent with our results and suggests that *cyp26b1* may be involved in the regulation of sex differentiation in *M. nipponense*. During gonad development, the transcription factor Steroidogenic Factor 1 (*SF1*) and Sex-Determining Region Y-Box 9 (*Sox9*) positively regulate *Cyp26b1* transcription, allowing for RA degradation and blocking germ cell differentiation in response to RA. This indicates that the Retinol metabolism pathway may be the key pathway for sex reversal in *M. nipponense*. Therefore, we suggest that E_2_ may affect hormone levels in the gonads by interfering with the expression of key genes in the hormone production pathway, thereby reversing male shrimp that are in the critical period of sex differentiation into female prawns.

In FM vs. NRM, several pathways are enriched, including Nucleotide excision repair (NER), DNA replication, Ribosome biogenesis in eukaryotes, Apoptosis-multiple species, and Homologous recombination (HR). NER is a major DNA repair pathway that eliminates various helix-distorting DNA lesions generated mainly by environmental mutagens such as ultraviolet light (UV) irradiation [22]. DNA replication regulates progress through the cell cycle as well as transcription, apoptosis, DNA repair/recombination, and DNA replication itself [23]. Ribosome biogenesis has been clearly linked to disease, particularly to cancer and anemia, and also to aging [24]. The apoptotic pathway regulates the highly specific and efficient construction, maintenance, and repair of redundant, misplaced or damaged cells [25]. HR serves to eliminate deleterious lesions such as double-stranded breaks and interstrand crosslinks from chromosomes [26]. It indicates that E_2_ induces DNA damage, interferes with the initiation of cellular transformation and leads to genomic instability. This is consistent with a study in zebrafish [27] in which E_2_ caused DNA damage and genomic instability with alterations in genes controlling ribosome synthesis. This implies that the sex reversal caused by E_2_ could damage male health. A high dose of E_2_ caused a significant reduction in male prawn sperm and some studies have shown that the effect of the hormone on sperm is related to oxidative DNA damage [28]. In particular, several studies have demonstrated that certain proteins of the NER pathways work cooperatively in the removal of oxidative lesions [29]. Therefore, it is suggested that NER may be an important pathway that estrogens inhibit sperm maturation. However, the reasons for their failure in sex reversal have not been found. A reasonable speculation is that with the growth of prawns, their own endogenous hormones gradually take a dominant role and influence the development of their secondary sexual characteristics [30]. Further refinement of feeding periods and doses may be needed to fully feminize the population of this species.

In this study, a total of 25 reproduction-related genes were screened in M vs. FM, M vs. RM, and FM vs. NRM. Some of these showed different gene expression patterns. Sperm gelatinase (*SG*) may play an important role in the regulation of sperm motility [31] (hyperactivation), acrosome reaction [32], sperm–egg fusion [33], and many other reproductive functions. *Mn-SG* is specifically expressed in the testes of *M. nipponense* and its expression level gradually increases with the degree of testis development. The level of SG decreases after RNA interference (RNAi)-induced knockdown of *Mn-SG* [34]. There were no SG screened in FM vs. NRM, further proving the reliability of the slice results. In addition, cathepsin C (*CatC*), heat shock protein cognate (*HSP*) and double-sex (*Dsx*) were specifically expressed in M vs. RM. *CatC* plays a central role in ovarian development in insects and fish and is thought to be an effective target for inhibiting rapid sexual maturation in female *M. nipponense* [35]. *HSP* is expressed under multiple environmental stressors to protect aquatic organisms and is found to be most highly expressed in stage IV of ovarian development in *M. nipponense,* playing a key role in regulating yolk synthesis [36,37,38]. *Dsx* is detected in the fruit fly (*Drosophila melanogaster*) sex determination cascade and is thought to play a negative feedback regulatory role in male *M. nipponense* development [39]. It means that these genes may be involved not only in the development of gonads but also in the process of sexual differentiation. Their specific expression proved the possibility of neo-males developing mature ovaries, confirming the reliability of histological observations. These results provide an important basis for regulating sex differentiation with E_2_ and establishing monoculture in juvenile *M. nipponense*. It is noteworthy that the gonadotropin-releasing hormone receptor (*GnRH*) was not screened for in M vs. RM. *GnRH* is a sex hormone that stimulates the synthesis and release of pituitary gonadotropins, playing a central role in controlling reproductive function in vertebrates [40,41,42]. In a study of rats, it was found that the majority of rats with removed testes were still able to produce some sperm after treatment with exogenous hormones [43]. This result indicated that the neo-males may not be able to produce endogenous estrogens on their own [30].

## 4. Materials and Methods

### 4.1. Experimental Prawns

Healthy pregnant female *M. nipponense* (body weight = 4.02 ± 0.55 g) were obtained from Taihu Lake (Wuxi, China; 120°13′4″ E, 31°28′22″ N) and maintained in a 500-L tank with a dissolved oxygen content of ≥ 6 mg/L at room temperature (28 ± 1 °C). The juvenile prawns hatched from these females were also cultured under the same condition.

### 4.2. Dietary Preparation

The diets used in this study were commercial prawn feed produced by Guangzhou Liyang Aquatic Products Co., Ltd. (Guangzhou, China). The commercial diet is mainly composed of crude protein, fish meal, shrimp meal, squid meal, starch, soybean meal, ash, canola meal, soybean protein concentrate, crude lipid, etc. [44]. The E_2_ (CAS number: 50-28-2, purity: 95.88%) was purchased from Beijing Solarbio Technology Co., Ltd. (Beijing, China). The method of dissolving E_2_ into the diets is described below [45]. E_2_ was dissolved in 95% ethanol to prepare a stock solution at a concentration of 50 mg/mL, then diluted into concentrations of 5, 10, and 20 mg/mL. Different concentrations of ethanol were then evenly sprayed on the feed (1 mL ethanol per 10 g diet) and stirred with a glass stick for at least 3 min. After that, it was placed under a ventilated laboratory hood and left in the shade for 15 min. The treated diets were added to 15 mL test tubes and placed in a refrigerator at 0 °C to evaporate the remaining alcohol naturally.

### 4.3. Experimental Design

**Figure 9:**
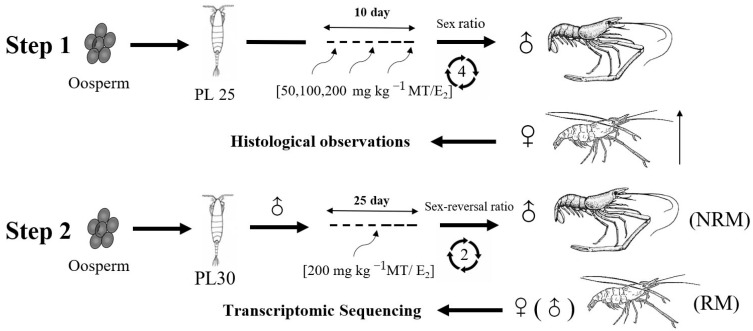
Experimental design. PL: post-larvae developmental stage.

The experimental design is shown in Figure 9. In the first step of the experiment, larvae were fed with *Artemia* until their body weight reached PL25 (0.0434 ± 0.0002 g). Diets with different concentrations of E_2_ were fed twice per day (at 8:00 and 20:00) at 2% of total body weight [46]. Three replications of each experimental group were made, each containing 200 juvenile prawns. The optimum concentration was determined by statistical sex ratio (female:male) and histological observation. Thereafter, the same method was used to feed larvae to PL30 (0.1281 ± 0.0002 g). Male prawns were selected and fed E_2_ for 50 days. Male prawns (M), female prawns (FM), neo-male prawns (RM), and unsex-reversed male prawns (NRM) were selected for transcriptome sequencing.

### 4.4. Determine Sex Reversal Concentration

#### 4.4.1. Sex Ratio Statistics

The prawns in the control and experimental groups were randomly selected from more than 90 individuals at 10, 20, 30, and 40 days, respectively, to determine the sex ratio. Each group had at least three replicates.

#### 4.4.2. Histological Observations

Females of *M. nipponense* were separated after being treated with different concentrations of E_2_ and stained with Hematoxylin and Eosin (HE) to study the histological changes in the ovary. After 40 days, samples of female prawns tested in different groups were mounted on slides and stained with HE and operated as described in previous studies [47]. Observed using a stereo microscope (SZX16; Olympus Corporation, Tokyo, Japan). Comparative labeling was performed with various cell types based on cell morphology [48].

### 4.5. Transcriptomic Sequencing

#### 4.5.1. RNA Isolation, Library Construction, and Sequencing

The gonads (male: testis; female: ovary) were collected from three individuals in each group and immediately stored in liquid nitrogen at a temperature of −190 °C. Total RNA was extracted by homogenizing the gonads with TRIzol reagent (Autolab Tech, Beijing, China). The RNA concentration was measured using a Qubit RNA Kit in conjunction with a Qubit 2.0 Fluorometer (Life Technologies, Carlsbad, CA, USA). Additionally, the purity of the RNA was evaluated utilizing a Nanodrop 2000 spectrophotometer (Thermo Scientific, Waltham, MA, USA). The integrity of the RNA was assessed employing an RNA Nano 6000 detection kit (2100 Bioanalyzer System; Agilent Technologies, Santa Clara, CA, USA).

A sequencing library was prepared using the NEBNext Ultra RNA Library Prep Kit (Illumina, San Diego, CA, USA) following the manufacturer’s instructions, with 3 µg of RNA from each sample. The RNA was purified and fragmented into small random pieces using poly-T oligo-attached magnetic beads (Life Technologies, Carlsbad, CA, USA). Double-stranded DNA was then synthesized using a TruSeq™ Stranded mRNA Prep Kit (Illumina). DNA fragments in the library with a length range of 150–200 bp were screened and purified utilizing an Ampure XP system (Beckman Coulter, Beverly, MA, USA). The purified double-stranded cDNA underwent size selection and connection before incubation with 3 mL of USER enzymes (NEB, Ipswich, MA, USA) at 37 °C for 15 min followed by culturing at 95 °C for 5 min. Polymerase chain reaction (PCR) was performed using Phusion High-Fidelity DNA polymerase, universal PCR primers, and index (X) Primer. The products were subsequently purified using the Ampure XP system. Finally, the composite samples were paired and sequenced using a HiSeq™ 25,000 for 2 × 100 bp according to the manufacturer’s instructions. Each lane’s PE reading was approximately 150 m (n = 3).

#### 4.5.2. Assembly and Dataset Annotation

The Illumina HiSeq high-throughput sequencing platform, which utilizes sequencing by synthesis technology, can generate a substantial amount of high-quality raw data. FastQC tools were used to truncate adapter and primer sequences and remove reads with N > 10% and quality (Q) < 5 for >50% of reads. Reads were assembled using Trinity according to the parametric transcription group, employing a minimum contig length of 300 and K-mer set at 27. To annotate the final set of unigenes comprehensively, BLAST software (https://blast.ncbi.nlm.nih.gov/Blast.cgi/, accessed on 26 February 2023) was used for comparing them with various databases such as NR (ftp://ftp.ncbi.nih.gov/blast/db/, accessed on 26 February 2023), Swiss-Prot (http://www.uniprot.org/, accessed on 26 February 2023), GO (http://www.geneontology.org/, accessed on 26 February 2023), COG (http://www.ncbi.nlm.nih.gov/COG/, accessed on 26 February 2023), KOG (http://www.ncbi.nlm.nih.gov/COG/, accessed on 26 February 2023), and KEGG (http://www.genome.jp/kegg/, accessed on 26 February 2023) databases (E-value ≤ 10^−5^). The amino acid sequences of unigenes were predicted and compared with the Pfam database using HMMER software (http://hmmer.org/, accessed on 26 February 2023).

#### 4.5.3. DEG Analysis and Quantitative Analysis

Six pairwise comparative sets of differentially expressed genes (DEGs) were obtained for M, FM, RM, and NRM groups using DESeq2 analysis. The false discovery rate (FDR) [49] was calculated using the Benjamini–Hochberg correction method to correct the significance of the *p*-values, with screening criteria of |log2(fold change)| ≥ 1 and FDR < 0.05 used to define DEGs. Pathway enrichment analysis of DEGs was performed using GO, COG, and KEGG annotation methods, with significantly enriched pathways identified based on a q-value < 0.05.

For the evaluation of sequencing and data analysis, qRT-PCR was performed to validate the DEGs. The gonad RNA was extracted (100 mg) using 1 mL TRIzol reagent (TaKaRa, Shiga, Japan), and first-strand cDNA synthesis was carried out through Reverse Transcriptase M-MLV Kit (TaKaRa). The qRT-PCR was performed using Bio-Rad iCycler iQ5 real-time PCR system (Hercules, CA, USA), with eukaryotic translation initiation factor 5 A as the reference gene [50]. The primers used are shown in Table S1. The reaction was amplified with 35 cycles at 94 °C for 30 s, 50 °C for 30 s, and 72 °C for 1 min, followed by 10 min incubation at 72 °C as a final extension step [51]. Each sample had four replicates while each reaction had three controls: nuclease-free water; primer-free water; and template-free water. The system recorded fluorescence curves and data automatically, and dissociation curves of the amplified products were analyzed at the end of each PCR. The mRNA expression levels were determined using the 2^−ΔΔCT^ method [52].

## 5. Conclusions

In conclusion, this study successfully induced sex reversal in *M. nipponense* through dietary supplementation of E_2_ and established the gonadal transcriptome of neo-males and unreversed males. The results showed that E_2_ can be used as a promising approach for sex reversal, but caution must be taken to prevent excessive hormone exposure. Furthermore, histological and transcriptional differences in the gonads of neo-males and unreversed males were compared, revealing key metabolic pathways and genes involved in sexual development. Retinol metabolism and the Nucleotide excision repair pathway were predicted to play an important role in sex reversal and sperm maturation in *M. nipponense*. Moreover, neo-males can develop normally and may not be able to produce endogenous estrogens on their own. These findings provide an important basis for regulating sex differentiation with E_2_ and establishing monoculture in juvenile *M. nipponense*. Further research may be needed to refine feeding times and doses to achieve complete feminization of the population.

## Figures and Tables

**Table 1:** Quality control and data statistics for clean reads.

Sample	Read Sum	Base Sum	GC (%)	Q20 (%)
M1	21,717,537	6,515,261,100	43.42	96.91
M2	19,968,322	5,990,496,600	43.19	97.04
M3	23,125,728	6,937,718,400	43.18	97.00
M4	24,640,844	7,392,253,200	43.83	96.64
FM1	21,357,638	6,407,291,400	43.00	96.88
FM2	23,085,893	6,925,767,900	42.96	96.86
FM3	21,412,841	6,423,852,300	43.27	96.72
FM4	18,150,368	5,445,110,400	42.71	96.82
RM1	23,917,031	7,175,109,300	41.40	96.95
RM2	22,740,630	6,822,189,000	43.41	96.70
RM3	24,818,214	7,445,464,200	42.96	96.54
RM4	25,021,439	7,506,431,700	43.52	96.38
NRM1	23,205,568	6,961,670,400	43.61	96.98
NRM2	25,279,785	7,583,935,500	43.89	96.45
NRM3	21,908,658	6,572,597,400	44.62	97.09
NRM4	19,861,207	5,958,362,100	44.75	96.64

**Table 2:** Differentially expressed pathways of three comparison libraries.

No.	Pathway ID	Pathway	Number of DEGs	q-Value
male prawns (M) vs. female prawns (FM)				
1	map02010	ABC transporters	14	0.0170
2	map04142	Lysosome	39	0.0170
3	map00520	Amino sugar and nucleotide sugar metabolism	25	0.0170
4	map00511	Other glycan degradation	13	0.0616
5	map03430	Mismatch repair	9	0.0652
6	map00981	Insect hormone biosynthesis	9	0.0703
7	map00900	Terpenoid backbone biosynthesis	6	0.0970
8	map00052	Galactose metabolism	9	0.0970
9	map00531	Glycosaminoglycan degradation	10	0.0970
male prawns (M) vs. neo-male prawns (RM)				
1	map04142	Lysosome	50	0.0000
2	map00981	Insect hormone biosynthesis	13	0.0001
3	map00520	Amino sugar and nucleotide sugar metabolism	25	0.0021
4	map00830	Retinol metabolism	15	0.0095
5	map02010	ABC transporters	11	0.0862
female prawns (FM) vs. unsex-reversed male prawns (NRM)				
1	map03420	Nucleotide excision repair	22	0.0003
2	map03030	DNA replication	20	0.0010
3	map03008	Ribosome biogenesis in eukaryotes	29	0.0013
4	map03022	Basal transcription factors	17	0.0076
5	map04215	Apoptosis—multiple species	12	0.0097
6	map03440	Homologous recombination	18	0.0097
7	map00520	Amino sugar and nucleotide sugar metabolism	29	0.0114

**Table 3:** Expression of reproduction-related genes in the transcriptome.

No.	Name	Accession Number	Up or Down		
			M/FM	M/RM	FM/NRM
1	sperm gelatinase	AFM38794.1	down	down	
2	doublesex and mab-3 related transcription factor	QDE10512.1	down	down	up
3	cyclin B	ADB44902.1	up	up	down
4	cystatin	AXS76129.1	up	up	down
5	cathepsin B	AUG69383.1	up	down	
6	cathepsin C	ROT62942.1		down	
7	cathepsin L	AHW49157.1		down	up
8	VASA-like protein	AEQ19569.1	up	up	down
9	heat shock protein cognate	AKB96209.1		down	
10	vitellogenin	AJP60219.1	up	up	down
11	vitellogenin receptor	AJP60220.1	up	up	down
12	ferritin	QDA69873.1	up	up	down
13	Fem1b	ANN47504.1	up	up	down
14	feminization-1	ALE66150.1	up	up	down
15	Kazal-type protease inhibitor	AEW24505.1	down	down	up
16	chitinase 3C	AHL28108.1	down	down	up
17	chitinase 1B	AHL28105.1	down	down	up
18	chitinase 3A	AHL28106.1	down	down	up
19	double-sex	QDE10516.1		down	
20	legumain-like protein	AJG06865.1		down	up
21	gametocyte-specific factor	AMY62701.1	up	up	down
22	gonadotropin-releasing hormone receptor	AHB33640.1	up		down
23	male reproductive-related protein	ABQ41234.1	down	down	up
24	peritrophin	ADB44903.1			up
25	gustavus	ADK46867.1			down

## Data Availability

The data presented in this study are available on request from the corresponding author for scientific purposes.

## Supplementary Materials

The following supporting information can be downloaded at: https://www.mdpi.com/article/10.3390/ijms24108481/s1.
